# Mindfulness, Gut–Brain Axis, and Health-Related Quality of Life: The Paradigm of IBD Patients

**DOI:** 10.3390/healthcare12121209

**Published:** 2024-06-17

**Authors:** Efstratios Christodoulou, Tsambika Mpali, Maroula-Eleni Dimitriadou, Antonios E. Koutelidakis

**Affiliations:** Laboratory of Nutritional and Public Health, Department of Food Science and Nutrition, University of the Aegean, 81400 Myrina, Greece; fnsd22002@aegean.gr (E.C.);

**Keywords:** mindfulness, gut–brain axis, health-related quality of life, inflammatory bowel disease, Mediterranean diet, well-being

## Abstract

Health-related quality of life (HRQoL) is a comprehensive measure that evaluates an individual’s well-being across physical, mental, and social dimensions. Enhancing HRQoL, particularly in individuals with chronic conditions like inflammatory bowel diseases (IBD), necessitates a holistic approach. Mindfulness, a scientifically supported strategy for managing anxiety, has shown promise in improving both physical and mental health. Its benefits may be partly explained through its effects on the gut–brain axis (GBA), a bidirectional communication link between the gastrointestinal system and the central nervous system. By exploring the interplay between mindfulness and the GBA, this study aims to uncover how these elements collectively influence HRQoL in both healthy individuals and those with IBD, offering insights into potential therapeutic pathways. A cross-sectional investigation involved 338 adults, including 50 IBD patients, utilizing validated Greek scales for Mindfulness (MAAS-15), Mediterranean Diet (14-MEDAS), and HRQoL (EQ-5D-5L). The questionnaire gathered demographic, anthropometric, and lifestyle data. Among healthy participants, EQ-5D-5L showed a moderate correlation with the MAAS-15 scale (r = 0.389, *p* < 0.05) and a low correlation with 14-MEDAS (r = 0.131, *p* < 0.05). IBD patients exhibited significantly lower mean EQ-5D-5L scores than healthy individuals (0.75 vs. 0.85, *p* < 0.05). MAAS-15 demonstrated a robust correlation (r = 0.414, *p* < 0.001) with EQ-5D-5L in IBD patients. Elevated mindfulness levels emerged as predictive factors for higher HRQoL in IBD patients (OR: 1.101, 95% CI: 1.008, 1.202, *p* < 0.05, compared to low mindfulness). In summary, factors influencing the GBA, including mindfulness and the Mediterranean diet, exhibit positive associations with HRQoL. Increased mindfulness levels predict better HRQoL in IBD patients, emphasizing the potential for clinical trials to validate these cross-sectional study findings.

## 1. Introduction

Modern lifestyles, marked by fast-paced living and numerous stressors, have heightened the focus on holistic well-being [1]. Neglecting the balance between physical and mental health has led to an increase in chronic conditions and a decline in quality of life [2]. Researchers are increasingly exploring the mind–body connection to develop strategies for lasting health. Psychoneuroimmunology has revealed significant links between psychological states, immune function, and overall health [3]. Within this field, the gut–brain axis (GBA) has emerged as crucial in understanding the interplay between mental well-being and physical health [4].

The GBA, a complex communication network, connects the central nervous system with the enteric nervous system in the gastrointestinal tract, facilitating bidirectional communication. This interaction affects cognitive and emotional processes and regulates gastrointestinal function [5]. This relationship extends to nutrition, psychology, and overall health, making the synergy between mindfulness and the GBA a key area of research with implications for health-related quality of life (HRQoL) [6].

Mindfulness, rooted in ancient contemplative practices, has gained scientific attention for its benefits in mental well-being and stress reduction [7]. Exploring the intersection between mindfulness and the GBA can provide insights into how present-moment awareness influences psychological states, gut microbiome balance, and gut function. Nutrition plays a significant role here, as dietary patterns affect both physical and mental health through their impact on the gut microbiota [8]. An example of a prudent dietary pattern that has been extensively researched and found to be beneficial for both gut microbiota as well as overall physical and mental health, is the Mediterranean diet. The Mediterranean diet is a nutritional pattern inspired by the traditional eating habits of countries bordering the Mediterranean Sea, emphasizing fruits, vegetables, whole grains, fish, and olive oil while minimizing red meat and processed foods. It is associated with various health benefits, including reduced risk of heart disease and improved overall well-being [9]. The Mediterranean diet and lifestyle share a common philosophical basis with mindfulness, as they both encompass values such as eudaemonia, sustainability, and sociability [10].

As researchers delve into the interconnections among mindfulness, nutrition, and the GBA, a compelling narrative emerges, suggesting that interventions targeting these intertwined elements may hold the key to enhancing health-related quality of life [11]. Health-related quality of life (HRQoL) serves as a comprehensive metric encompassing the multidimensional aspects of an individual’s well-being, reflecting not only the absence of disease but also the intricate interplay among physical health, mental states, and social functioning [12]. 

This study focuses on HRQoL in individuals with inflammatory bowel disease (IBD), a chronic disorder that affects the digestive tract and impacts daily life and social interactions [13]. Understanding and improving HRQoL in IBD patients involves exploring the roles of mindfulness and the GBA in their overall well-being. The aim is to investigate the dynamics influencing HRQoL in both healthy individuals and IBD patients. By examining the convergence of mindfulness, nutrition, and the GBA, the study seeks to contribute to holistic approaches that improve quality of life and offer new therapeutic avenues for those dealing with complex health conditions.

## 2. Materials and Methods

### 2.1. Design and Procedure

This research employed a cross-sectional research design, utilizing an online survey administered in both the Greek and English languages via the Sogolytics online survey tool [14]. The initial two survey questions, regarding the acceptance of the study’s terms and conditions and the verification of their age within the 18-to-65-years range, served as inclusion and exclusion criteria. The survey was distributed through direct messaging on various social media platforms, accompanied by follow-up reminders to foster participant engagement and establish a platform for addressing any inquiries or concerns related to the study. The survey was also forwarded to the online platforms of the Hellenic Society of Crohn’s Disease and Ulcerative Colitis’ Patients. This hybrid approach capitalizes on the advantages of both web-based and e-mailed questionnaires [15,16,17].

The research objectives and hypotheses were fully stated before beginning the data collection process, offering a comprehensive framework for the subsequent analysis and interpretation of the research findings. The final sample that satisfied the inclusion–exclusion criteria comprised a total of 315 adults.

### 2.2. Scales

#### 2.2.1. Mindfulness Awareness Attention Scale (MAAS-15)

The Mindful Attention Awareness Scale (MAAS-15) stands as a robust and widely accepted tool for quantifying mindfulness [18]. Developed by Kirk Warren Brown and Richard M. Ryan, this self-report measure targets an individual’s receptive awareness of and attention to present-moment experiences [19]. Spanning 15 items, the MAAS captures the essential facets of mindfulness, emphasizing attention and awareness in everyday situations. Its design diverges from conventional mindfulness scales by probing into the innate capacity for sustained attention across various activities rather than focusing solely on meditation or specific mindfulness practices. This inclusive approach renders the MAAS versatile, offering a comprehensive assessment applicable to diverse contexts and populations. Its psychometric properties contribute to its credibility as a reliable measure; the scale demonstrates high internal consistency, test–retest reliability, and construct validity, aligning well with the theoretical underpinnings of mindfulness. Moreover, the MAAS avoids jargon and convoluted language, making it accessible and user-friendly for respondents, irrespective of their familiarity with mindfulness concepts. Its widespread use across cultural and demographic boundaries attests to its transcultural applicability, a crucial aspect in measuring such subjective experiences [20]. The MAAS-15 scale is validated in Greek [21]. In our study MAAS-15 had a reliable internal consistency index (Cronbach’s α), α = 0.896

#### 2.2.2. Mediterranean Diet Adherence Scale (14-MEDAS)

Schroeder et al. [22] developed the 14-MEDAS scale as part of the PREDIMED trial framework. It is a 14-item instrument for assessing adherence to the Mediterranean diet. A sample question from the 14-MEDAS is “how much olive oil do you consume in a given day?” Each query is scored on a scale of 0 to 1, and the total 14-MEDAS score ranges from 0 to 14 points. Low adherence is indicated by scores ranging from 0 to 5, moderate adherence by 6 to 9, and high adherence by 10 to 14 (22). In a study by García-Conesa et al. [23], the Greek version of 14-MEDAS had a significant concordance (81.2 ± 10.7%) with the Food Frequency Questionnaire (FFQ), indicating its validity and reliability as a research instrument for assessing Mediterranean diet adherence in the Greek population.

#### 2.2.3. The EuroQol 5-Dimension 5-Level Questionnaire (EQ-5D-5L)

The EQ-5D-5L scale serves as a pivotal instrument in assessing health-related quality of life (HRQoL), offering a comprehensive and multidimensional evaluation. Developed by the EuroQol Group, this standardized measure evaluates health status across five domains: mobility, self-care, usual activities, pain/discomfort, and anxiety/depression. What sets the EQ-5D-5L apart is its simplicity and ease of administration; respondents rate their health state on each dimension using a five-level scale, capturing a more nuanced and refined assessment compared to its predecessor, the EQ-5D-3L. Its improved granularity allows for a more precise quantification of health states, enhancing sensitivity to subtle changes in health conditions and treatment outcomes [24]. Furthermore, the EQ-5D-5L is validated in Greek [25]. Its versatility and brevity make it suitable for various applications, including clinical trials, population health surveys, and economic evaluations in healthcare. This standardized measure facilitates cross-comparisons across different health conditions and interventions, enabling healthcare professionals, researchers, and policymakers to make informed decisions based on a robust assessment of HRQoL [26]. Despite its widespread use and applicability, the EQ-5D-5L does have limitations, such as its generic nature, which might not capture condition-specific nuances. Nonetheless, its ability to succinctly capture multidimensional aspects of HRQoL, its simplicity in administration, and its adaptability across diverse populations cement its position as an indispensable tool in assessing health outcomes and guiding healthcare interventions [27]. The score of the EQ-5D-5L scale (EQIndex) is calculated by converting responses on the five dimensions of health (mobility, self-care, usual activities, pain/discomfort, and anxiety/depression) into a single index using country-specific value sets, with a range typically between −0.594 and 1, where 1 represents full health and values less than 0 indicating health states worse than death [28].

### 2.3. Demographic and Anthropometric Information

Demographic and somatometric data items were positioned toward the end of the survey in order to reduce survey dropout rates [29]. The demographic questions included aspects like gender, work status, education, and marital status. Participants were asked to submit their weight and height in order to have their Body Mass Index (BMI) calculated. Although self-reported height and weight may not provide the most precise information about the respondents’ body composition, this method is nonetheless useful for determining BMI in adult populations from a variety of sociodemographic backgrounds [30]. Individuals’ BMIs were divided into subcategories, such as underweight, normal weight, overweight, and obesity, based on established norms [31].

### 2.4. Data Analysis

A comprehensive review of the data was performed to detect any potential omissions. Data from participants who abruptly discontinued the questionnaire (considered Missing Completely at Random) were excluded from the analysis [32]. For data points missed accidentally (classified as Missing at Random), the missing values were replaced with the mean of all the respondents’ answers.

The data were exported in a format suitable for SPSS v28 for import and processing. Statistical analysis and visualization were carried out using SPSS v28. Before conducting statistical tests, a regularity check was performed to ensure the data distribution met the required criteria. The literature suggests conducting a regularity test prior to statistical analyses. To achieve the most accurate assessment of regularity, both visual inspection and the Shapiro–Wilk test were used [33]. 

Statistical analyses included Pearson’s correlation, independent samples t-test, and one-way ANOVA for continuous variables that followed a normal distribution, as determined by the Kolmogorov–Smirnov test. Multinomial logistic regression was used to evaluate the impact of mindfulness on HRQoL in individuals with IBD, with adjustments for potential confounders. The significance level for statistical tests was set at *p* < 0.05.

## 3. Results

### 3.1. Subjects

The survey initially included 376 participants. After removing 35 respondents who did not meet the inclusion criteria, along with three respondents who provided incomplete (answered less than 50% of the questions) or random responses (completed in under 3 min), the final sample consisted of 338 individuals (75.4% female, 24.3% male, and 0.3% non-binary). Significant differences (*p* < 0.05) were found in the average scores of MAAS-15, MEDAS, and BMI across different age groups, with younger participants showing lower scores. A higher education level was associated with significantly higher MAAS-15 and MEDAS-14 scores (*p* < 0.05). Married participants had significantly higher (*p* < 0.05) MAAS-15 scores. 

### 3.2. Correlations between Mindfulness, the Mediterranean Diet, HRQoL and BMI among Healthy Individuals

Pearson’s correlation coefficients were used to evaluate the potential relationships among MAAS-15, 14-MEDAS, EQIndex, and BMI in healthy persons. The data analysis demonstrated a significant correlation between MAAS-15 and 14-MEDAS with EQIndex. Additionally, a negative correlation was observed between EQIndex and BMI (Table 1).

### 3.3. Correlations between Mindfulness, the Mediterranean Diet, HRQoL and BMI in Individuals with IBD

To explore potential relationships among MAAS-15, 14-MEDAS, EQIndex, and BMI in patients with IBD, Pearson’s correlation coefficients were calculated. Statistical analysis indicated significance only between MAAS-15 and EQIndex (Table 2). There was no statistically significant correlation among EQIndex, 14-MEDAS, and BMI.

### 3.4. HRQoL Assessments in Healthy Individuals and Individuals with IBD

**Figure 1:**
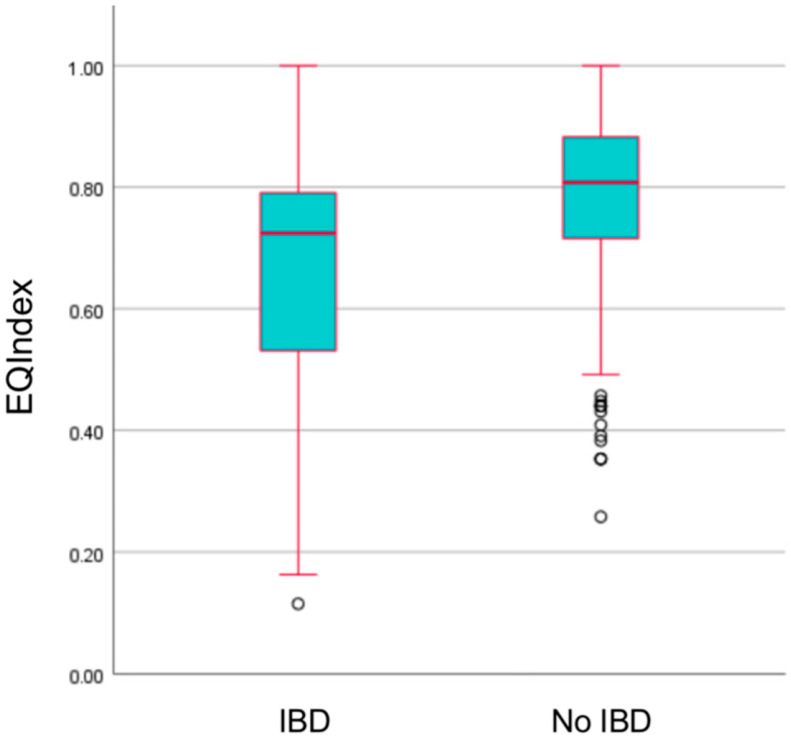
Boxplots depicting the statistically significant difference in HRQoL scores between IBD patients and healthy individuals.

The analysis of HRQoL assessments revealed notable distinctions between healthy individuals and those diagnosed with IBD. Individuals with IBD exhibited significantly lower HRQoL scores, as indicated by their EQIndex scores, compared to healthy counterparts (Figure 1). Furthermore, a comprehensive examination of the EQ-5D-5L scale dimensions revealed statistically significant differences between individuals with IBD and healthy individuals across all aspects, including mobility, self-care, usual activities, pain/discomfort, and anxiety/depression.

### 3.5. Mindfulness, the Mediterranean Diet and HRQoL

**Figure 2:**
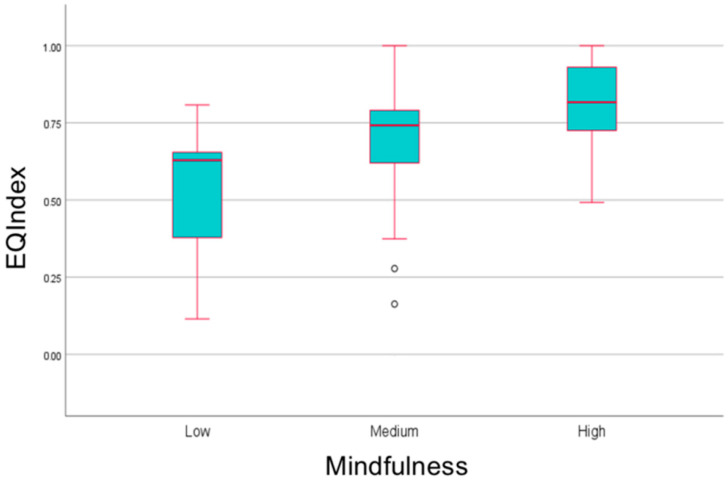
Boxplots of HRQoL scores in different mindfulness levels among individuals with IBD.

In our investigation, mindfulness demonstrated a moderate correlation (r = 0.365, *p* < 0.001) with HRQoL for healthy individuals, while mindfulness had a robust correlation (r = 0.414, *p* < 0.001) with HRQoL among individuals diagnosed with IBD (Figure 2). This finding highlights a stronger association between mindfulness and the perceived quality of health among IBD patients.

Moreover, the multinomial logistic regression analysis revealed that heightened levels of mindfulness served as predictive factors for improved HRQoL in individuals living with IBD (OR: 1.101, 95% CI: 1.008, 1.202, *p* < 0.05) when compared to those with lower levels of mindfulness (Table 3).

## 4. Discussion

The present study represents the first known investigation into the interconnections among mindfulness, the GBA, and HRQoL in Greece, specifically in individuals diagnosed with IBD. By focusing on this intersection, our research expands upon the existing literature concerning the psychological functioning of individuals with IBD [6]. While previous studies have examined various aspects of psychological well-being and coping strategies in this population [34], to our knowledge, none have explored the combined influence of mindfulness and factors affecting the gut–brain axis such as the Mediterranean diet on HRQoL in individuals with IBD.

The results of the study examining the association between mindfulness, the GBA, and HRQoL provide valuable insights into the interplay of psychological and physiological factors in influencing well-being, particularly in the context of IBD. The findings reveal several key observations. Firstly, among healthy participants, there is a moderate correlation between HRQoL and mindfulness, indicating that individuals with higher levels of mindfulness tend to report better overall well-being. This aligns with previous research demonstrating the positive impact of mindfulness on various aspects of health and quality of life [35,36,37]. Similarly, the low correlation between HRQoL and adherence to the Mediterranean diet suggests that while diet may influence certain dimensions of well-being, its association with overall quality of life may be less pronounced. These findings corroborate the existing literature, highlighting the complex relationship between dietary habits and HRQoL, with dietary patterns such as the Mediterranean diet often linked to improved health outcomes but with varying effects on overall well-being [38]. Studies suggest that dietary patterns high in fiber and low in processed foods can positively influence the gut microbiota, thereby enhancing gut health and potentially improving HRQoL [39]. Conversely, diets high in processed foods and sugars have been linked to dysbiosis and poorer health outcomes, further demonstrating the profound effect of diet on overall health and quality of life [40].

Individuals with IBD exhibit significantly lower mean HRQoL scores compared to healthy counterparts, underscoring the substantial impact of this chronic inflammatory condition on overall well-being. However, within the IBD population, higher levels of mindfulness are strongly correlated with better HRQoL. This novel finding suggests that mindfulness interventions may hold promise for mitigating the negative effects of IBD on quality of life. This aligns with previous research indicating the beneficial effects of mindfulness-based interventions on symptom management and coping strategies in individuals with chronic illnesses [41].

Furthermore, the study identifies elevated mindfulness levels as predictive factors for higher HRQoL in IBD patients, highlighting the potential therapeutic value of mindfulness-based interventions in clinical practice. This supports the hypothesis that enhancing mindfulness may lead to improvements in quality of life among individuals with chronic inflammatory conditions and is consistent with earlier studies. [42,43]. 

However, it is essential to interpret these findings within the broader context of the study’s limitations. The cross-sectional design limits the ability to establish causality or determine the direction of the observed associations. Additionally, the reliance on self-reported measures introduces the possibility of response bias and does not capture objective physiological markers of mindfulness or dietary adherence. Moreover, the study’s sample size may restrict the generalizability of the findings, warranting caution in extrapolating the results to broader populations.

Future research directions may include longitudinal studies to assess the long-term effects of mindfulness interventions on HRQoL in individuals with IBD, as well as randomized controlled trials to evaluate the efficacy of mindfulness-based interventions as adjunctive treatments for IBD. Additionally, investigations into the underlying mechanisms through which mindfulness influences the GBA and physiological pathways implicated in IBD could provide further insights into the potential therapeutic targets for intervention. 

## 5. Conclusions

This study underscores the intricate interplay between factors affecting the GBA, such as mindfulness practices and adherence to the Mediterranean diet, and their profound impact on HRQo. Notably, heightened levels of mindfulness emerge as a significant predictor of enhanced HRQoL among individuals grappling with IBD, suggesting a promising avenue for targeted interventions. The observed positive associations between GBA-influencing factors and HRQoL underscore the importance of further investigation through robust clinical trials to corroborate the findings gleaned from this cross-sectional study. Such trials hold the potential to illuminate the efficacy of mindfulness-based interventions as adjunctive therapies in ameliorating the HRQoL challenges posed by IBD, offering hope for improved patient outcomes and well-being in clinical settings. Overall, while the study provides promising insights into enhancing quality of life for individuals with IBD, further research is necessary to validate and build upon these findings.

## Figures and Tables

**Table 1:** Pearson’s correlations (r) among MAAS-15 (mindfulness), 14-MEDAS (Mediterranean diet), EQIndex (HRQoL), and BMI.

	MAAS-15	14-MEDAS	EQIndex	BMI
MAAS-15	1	0.080	0.389 **	0.007
14-MEDAS	0.080	1	0.131 *	−0.003
EQIndex	0.389 **	0.131 *	1	−0.208 **
BMI	0.007	−0.003	−0.208 **	1

* Correlation is significant at the *p* < 0.05 level. ** Correlation is significant at the *p* < 0.001 level.

**Table 2:** Pearson’s correlations (r) among MAAS-15 (mindfulness), 14-MEDAS (Mediterranean diet), EQIndex (HRQoL) and BMI in IBD patients.

	MAAS-15	14-MEDAS	EQIndex	BMI
MAAS-15	1	0.041	0.414 *	0.066
14-MEDAS	0.041	1	0.016	−0.011
EQIndex	0.414 *	0.016	1	−0.137
BMI	0.066	−0.011	−0.137	1

* Correlation is significant at the *p* < 0.001 level.

**Table 3:** Multinomial logistic analysis results.

	OR	95% CI	*p*-Value
Mindfulness (Low vs. High)	1.101	1.008–1.202	<0.05

## Data Availability

The data presented in this study are available upon request from the corresponding author.
